# Summit Ties Sustainability to Improved Public Health

**DOI:** 10.1289/ehp.120-a19

**Published:** 2012-01-01

**Authors:** Tanya Tillett

**Affiliations:** Tanya Tillett, MA, of Durham, NC, is a staff writer/editor for *EHP*. She has been on the *EHP* staff since 2000 and has represented the journal at national and international conferences.

Armed with enthusiasm, expertise, and a desire for sustainable solutions to meet the current and future needs of society, participants met 7–8 November 2011 in Research Triangle Park, North Carolina, for the fourth annual summit of the Research Triangle Environmental Health Collaborative, titled “Incorporating Public and Environmental Health into Sustainable Solutions.”^1^

Over the course of the 2-day summit, the attendees discussed and fine-tuned a list of suggestions addressing the 3 pillars of the sustainability paradigm as defined by the United Nations^2^—society, environment, and economy. Suggestions revolved around finding concrete ways to connect the cost savings associated with sustainable products and services to improved public health outcomes, communicating with the public and engaging it in sustainability decision making, and focusing on long-term creation of a built environment that supports healthier people. The final recommendations will be presented by the Collaborative to policy makers on the local, state, and national levels and submitted to a peer-reviewed journal for publication.

Facing the reality of the gap between what the Earth can provide and what humanity needs has led to an increase in innovative initiatives in the public and private sectors, said Jay Golden, director of the Duke Center for Sustainability and Commerce, although as he noted in his plenary speech, industry has been more aggressive than the public sector, mainly because of strong drivers that include rapid urbanization and expansion of the middle class worldwide, as well as regulatory frameworks, consumer demand, and the quest for market share. Sustainability has become a major selling point among environmentally conscious consumers.

Golden discussed some of the sustainable practices and programs being adopted by producers in the consumer product industry aimed at conserving resources, including sustainable production practices implemented by the Outdoor Industry Association^3^ as well as the creation of such manufacturing and industry entities as the Sustainable Apparel Coalition^4^ and the Sustainability Consortium.^5^ He also mentioned the National Science Foundation’s Sustainability Research Networks competition, which funds the development of new transdisciplinary research collaborations in sustainability science, engineering, and education.^6^

In his speech to summit participants, Adam Finkel, executive director of the University of Pennsylvania’s Penn Program on Regulation and a professor of environmental and occupational health at the University of Medicine and Dentistry of New Jersey School of Public Health, discussed the use of risk assessment information in a more targeted, tangible way to make better sustainability choices—in short, how to combine the best features of both risk assessment and the desire to make precaution- and sustainability-based decisions to create tangible, positive end results.

According to Finkel, classic risk assessment can be too analytical, focused solely on finding acceptable levels of exposures to potential harmful substances one at a time, while many sustainability programs can depend too heavily on intuition about what might improve public health and the environment. Public health can be improved by comparing the costs and benefits of possible alternatives instead of simply identifying acceptable exposure levels or risk reduction quotas, he said.

Also problematic, he added, is the lack of attention given by policy makers to workplace exposures. “People bearing the highest exposures are always those that make the products. . . . Sustainability programs are terrific, but they focus more on society as a whole and not on the well-being of the workers who are faced with [potentially] harmful exposures.” Instead of using risk assessment to identify acceptable levels of exposure to occupational chemicals, policy makers should be looking at safer alternatives that can be used hand-in-hand with sustainable methods. Alternative manufacturing processes based on this proposed combined methodology could better serve to protect the health of those who make products, not just the people who use them, Finkel said.

Lek Kadeli, deputy assistant administrator for management of the U.S. Environmental Protection Agency Office of Research and Development, discussed the need for a holistic approach to incorporating sustainability into the federal government. He acknowledged the challenges involved in implementing sustainability initiatives given current federal mandates and long-standing approaches to environmental and health protection. Kadeli noted that many of these challenges can be met by relying on more interdisciplinary and systems-based approaches such as interagency work groups and partnerships.

Michael McGeehin, former director of the Division of Environmental Hazards and Health Effects of the National Center for Environmental Health and current senior environmental health epidemiologist with the Climate Change Health Effects Program of the Research Triangle Institute, who also spoke at the summit, said the urban built environment is another piece of the puzzle that should be given more attention when it comes to connecting sustainability to improved public and environmental health.

“It’s a tenet of biology that all living organisms are impacted both positively and negatively by their environment,” McGeehin said. “Since most human beings and the vast majority of Americans live in cities, their environment is primarily the [urban] built environment. The buildings, streets, traffic, green spaces, food availability, safety, and many other aspects of day-to-day living have a direct effect on a person’s mental and physical well-being.” With nearly three-quarters of the world’s population projected to inhabit urban areas by 2050,^7^ and the majority of that urbanization occurring in developing countries,^8^ McGeehin says it will be more important than ever to base the built environment decision-making process on sound principles of sustainable development.
